# Examining the audiovisual therapy effects on hospital groups of varying linear canopy landscapes and those with hydrodynamic forces

**DOI:** 10.3389/fpubh.2024.1324260

**Published:** 2024-09-03

**Authors:** Ping Zhang, Yixin Cui, Ke Luo, Tongyao Zhang, Yanbin Yang, Jinpeng Li, Mingze Chen, Hao Chen, Qianyi He, Zheng Yu, Guangyu Wang, Xiaohua Wang, Weiquan Guo, Xi Li, Jun Ma

**Affiliations:** ^1^College of Landscape Architecture, Sichuan Agricultural University, Chengdu, China; ^2^Department of Internal Medicine, Wenchuan Traditional Chinese Medicine Hospital, Wenchuan, China; ^3^Department of Forest and Resources Management, Faculty of Forestry, University of British Columbia, Vancouver, BC, Canada; ^4^The 3rd People’s Hospital of Wuhou, Chengdu, China; ^5^Department of Internal Medicine, Sichuan Province Forestry Central Hospital, Chengdu, China

**Keywords:** visual-sound interaction, canopy landscape, landscape with medium and low hydrodynamic forces, therapeutic benefits, hospital groups

## Abstract

Recent research has highlighted the beneficial effects of urban green spaces on physical and mental health. This study focused on the hospital population and innovatively subdivided the population into four groups: doctors, caregivers, patients and nurses. A total of 96 volunteers participated in this virtual reality experiment to assess the restoration of a linear canopy landscape and a landscape with different levels of hydrodynamics through interactive audiovisual immersion. We utilized pre-research method, brainwave monitoring technique, psychological scales, observation and interviews in this experiment. The research identified five key findings. First, both linear canopy landscapes and those with low to medium hydrodynamic forces significantly enhance physiological and psychological restoration for all groups, with the most substantial physiological benefits observed in doctors and patients, and the greatest psychological relief noted in caregivers. Second, landscapes with medium hydrodynamic forces yield higher restorative effects than those with low forces in hospital settings. Third, green landscapes with medium and low-density canopies prove more conducive to patient recovery compared to those with high-density canopies. Fourth, the inclusion of bird songs does not markedly affect physiological restoration across the hospital groups. Finally, landscapes that incorporate elements of water dynamics, open skies, and lightly foliated canopies draw significant interest from all groups involved. This study advocates for the integration of natural blue and green elements into hospital environments as complementary therapeutic interventions, aiming to alleviate stress and promote health recovery among hospital communities.

## 1 Introduction

Individuals in hospitals can be divided into three key groups: patients; medical staff; and caregivers. According to the results of previous observations of the behaviors of the outdoor space users and the interviews on them, the patients experience negative emotions such as anxiety, irritability, pessimism, and loneliness due to illnesses. They long for a cure. Patients in short-term treatment need space to sit and rest, and inpatients want to socialize with others, move around, and keep in touch with nature. Healthcare workers face great mental stress with low energy. They are at a significantly high risk of suffering from mental disorders than the general population (1), and also need some space for relaxing and leisure. Family members accompanying the patients suffer from physical fatigue, loneliness, boredom, and anxiety. They provide short-term or long-term care and worry for the patients. They also crave care, and companionship in need of private spaces for solitude and conversations.

Exposure to and appreciation of nature has been shown to effectively reduce physical and mental stress (2), alleviate mental fatigue, and improve attention restoration (3, 4), concentration, and cognition (5). In addition, nature promotes positive emotions, reduces negative emotions (6, 7), and enhances mental health and well-being (8, 9). The effects of natural landscapes on health vary with the characteristics and compositions of the landscapes. For example, viewing natural tree formations can result in a more positive emotional experience and lower blood pressure than artificially pruned geometric tree formations (10). In urban parks or natural green spaces, tree canopies are a valuable natural feature, allowing visitors to have a relaxing experience while resting under them. Studies reported that visitors express feelings of happiness for the first time when lying down in forests and looking at the sky (11, 12). “canopy landscape” is defined as the sum of landscape elements in the entire upwards perspective. it was found that forest canopy landscapes with different trunk, leaf, and sky occupancy ratios give different experiences. Furthermore, green landscapes with light are highly restorative (13). A large amount of research has been conducted on both blue and green landscapes, revealing their role in reducing obesity rates (14) and the risk of cardiovascular and respiratory diseases (15), and promoting mental health (16). Water features have the power of spirituality to inspire, purify, and heal people. Mental health promotion benefits vary depending on the type of water feature; freshwater water features are mostly soft and inviting and can significantly reduce mental fatigue and psychological stress. Falling water landscapes produce the greatest promotional befits (17).

Natural landscapes have been introduced into the outdoor environment of hospitals as therapeutic landscapes, serving a remarkable restorative function as medical resources (18). Given the characteristics of the hospital population and the functional requirements of hospital settings, outdoor spaces in hospitals must have concise, visibly clear, and accessible design features. Unlike parks, the layout of outdoor green spaces in hospitals cannot adopt the same diversity in scale and type. Moreover, research indicates that linear canopy landscapes also have health-promoting effects (19), providing spaces for patients to observe, walk, and rest, thereby increasing accessibility and opportunities for green space visitations.

Vision and hearing are the two most important senses of human perception (20). Loudness and sound pressure level are the most significant features of acoustic perception (21). As a subjective measurement of human perception of sound intensity, loudness is influenced by several factors such as frequency and loudness level. Loudness can be described in terms of the measurement unit decibel (22, 23). The sound pressure level, the most basic acoustic indicator of the physical characteristics of a soundscape, is a physical quantity that directly reflects the magnitude and strength of the decibel values and is based on the human ear’s perception of sounds (24). The human ear above 30 can perceive sounds ranging from 20 to 20,000 Hz in frequency. The pressure level of water sounds is affected by numerous factors, including the water body flow velocity, the height of falling water, and the material of the underlay blasted by water (e.g., rocks, aquatic plants, water bodies, leaf litter, dead wood floats, etc.), and reveals differences in the water sound intensity. The sound sources of running-water soundscapes are mainly affected by the flow velocity of water. The present study considers the “hydrodynamic landscapes” the water landscapes with a certain flow rate, which produce sound pressure levels and sound intensity that can be perceived by human.

Natural environments are not accessible for everyone (25), particularly for hospital populations, due to the limited time and ability to leave the hospital (26). Virtual reality (VR) is considered to be highly similar to natural physical experiences (27, 28) and has been applied to restorative assessments, particularly in healthcare settings. Moreover, a combination of nature and VR in healthcare settings can be an effective alternative to analgesics, resulting in a reduction in additional medical applications (29). Therefore, VR can potentially be applied to improve mental health and reduce patients’ negative emotions, pain, and anxiety.

Two basic theoretical approaches are generally used for research on the restorative effects of nature experiences, namely, Attention Restoration Theory (ART) and Stress Reduction Theory (SRT). ART suggests that natural environments that are distant, extended, attractive, and compatible can promote recovery from attention fatigue (30), and SRT suggests that exposure to nature has a stress-relieving effect compared to urban landscapes (31). The Self-rating Restoration Scale (SRRS), another more targeted short modified version of the self-report scale, was developed by Ke-Tsung Han that covers emotional, physical, cognitive, and behavioral dimensions for assessing the degree of perceived recovery in different natural environments, and has been validated and utilized (32).

In this study, the hydrodynamic forces used to characterize water sounds are physical (sound pressure level) and perceptual (loudness) features. Since it is difficult to perceive sounds with a stable sound pressure level from 10 to 15 dB, the minimum threshold of the hydrodynamic gradient in this study is 30 dB. Low, moderate, and high sound levels of running water with low hydrodynamic forces are defined to range from 30 to 50 dB, 51 to 70 dB, and 71 dB and above, respectively. Due to the specificity of the test subjects, running water with high hydrodynamic forces is not involved in this study. Once the hydrodynamic gradient is defined, we investigate the following three key questions.

1. Can virtual reality (VR) audiovisual perceptions of linear canopy landscapes (green landscapes) and landscapes with low to medium hydrodynamic forces (blue landscapes) provide restorative benefits for hospital populations?
2. What are the differences in the physiologically and psychologically restorative benefits of these landscapes for different hospital populations?
3. What kind of composition contributes to the restorative benefits of the landscapes?

## 2 Methods

### 2.1 Participants

A total of 96 subjects (47 men and 49 women) participated in the study. Of these, 48 were health-care workers (internal medicine, paediatrics, obstetrics and gynaecology, and anorectal medicine) aged between 20 and 55 years, with a mean age of 31 years, and a mean monthly income of approximately US$ 1,013, 24 patients (some suffering from hypertension, cerebral embolism, cerebral infarction, encephalatrophy, coronary heart disease, depression, chronic renal failure, cancer, diabetes mellitus, pneumonia, and puerperal syndromes) aged from 6 to 70 years, with an average age of 46 years, and an average monthly income of approximately $719. 24 caregivers (with care spanning 7 days to 7 years) between 40 and 60 years of age, with an average age of 46 years, and an average monthly income of approximately $585. Prior to the experiment, vision and hearing tests and a color blindness test were conducted to ensure that all subjects had normal vision and hearing and no color recognition impairment. Information obtained prior to the experiment indicated that all volunteers were willing to visit green spaces, and their access to green spaces was influenced by factors such as accessibility, income, leisure time, and distance. The study was conducted in accordance with Declaration of Helsinki and is supported by the Ethics Committee of both 3rd People’s Hospital of Wuhou, Chengdu and Sichuan Province Forestry Center Hospital.

### 2.2 Stimulus material

**Figure 1:**
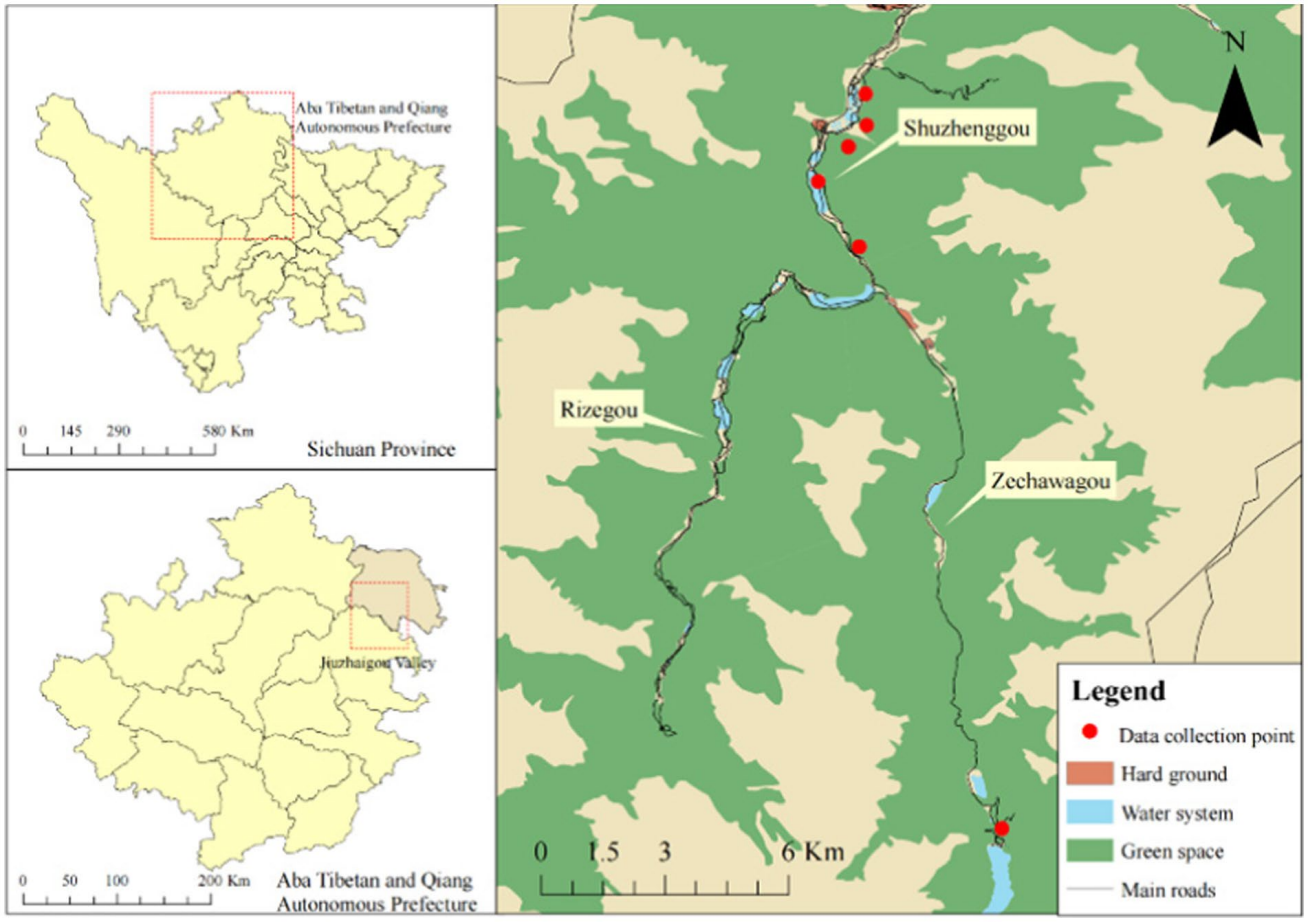
Location of the VR video collection sites.

Before the experiment, the study group collected VR videos from the Jiuzhaigou Valley (Figure 1) during 26–31 July 2022. The valley is a World Natural Heritage Site and a famous tour destination in China. Jiuzhaigou is rich in waterscape types, features, and compositions and is known for its variety of colorful forests, mountains, and snowy peaks (33). The scenic area is paved with 60 km of wooden sidewalks, along which there are a rich variety of landscapes, providing good conditions for the data collection and data screening of this study.

**Figure 2:**
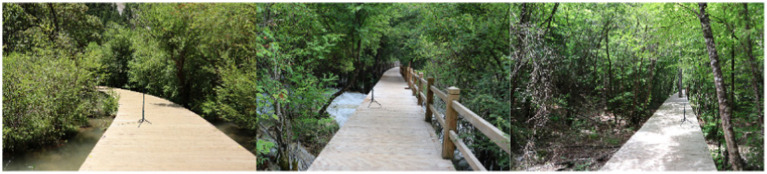
Example images from the panoramic videos taken in Jiuzhaigou Valley.

In this experiment, panoramic images were shot with an Insta 360 panoramic camera (15–20 mega-pixels and a 4/3-inch sensor, 360 Onex, Shenzhen) (Figure 2). Images were collected from 9 to 11 in the morning and from 2 to 4 in the afternoon to ensure similar weather conditions. The average daytime temperature humidity in Jiuzhaigou Scenic Spot for July 2022 was 30°C and 85%, respectively. For the collection of images, the camera was placed on a tripod with the lens at the same height as human eyes (1.2 m). A recorder (Zoom H5, Japan), an outdoor directional microphone (RODE NTG4, Australia), and a sound level meter (Aiwa 6228, Japan) were used to collect the data on nature sounds including water sounds and bird songs.

### 2.3 Visual-aural stimulation

**Figure 3:**
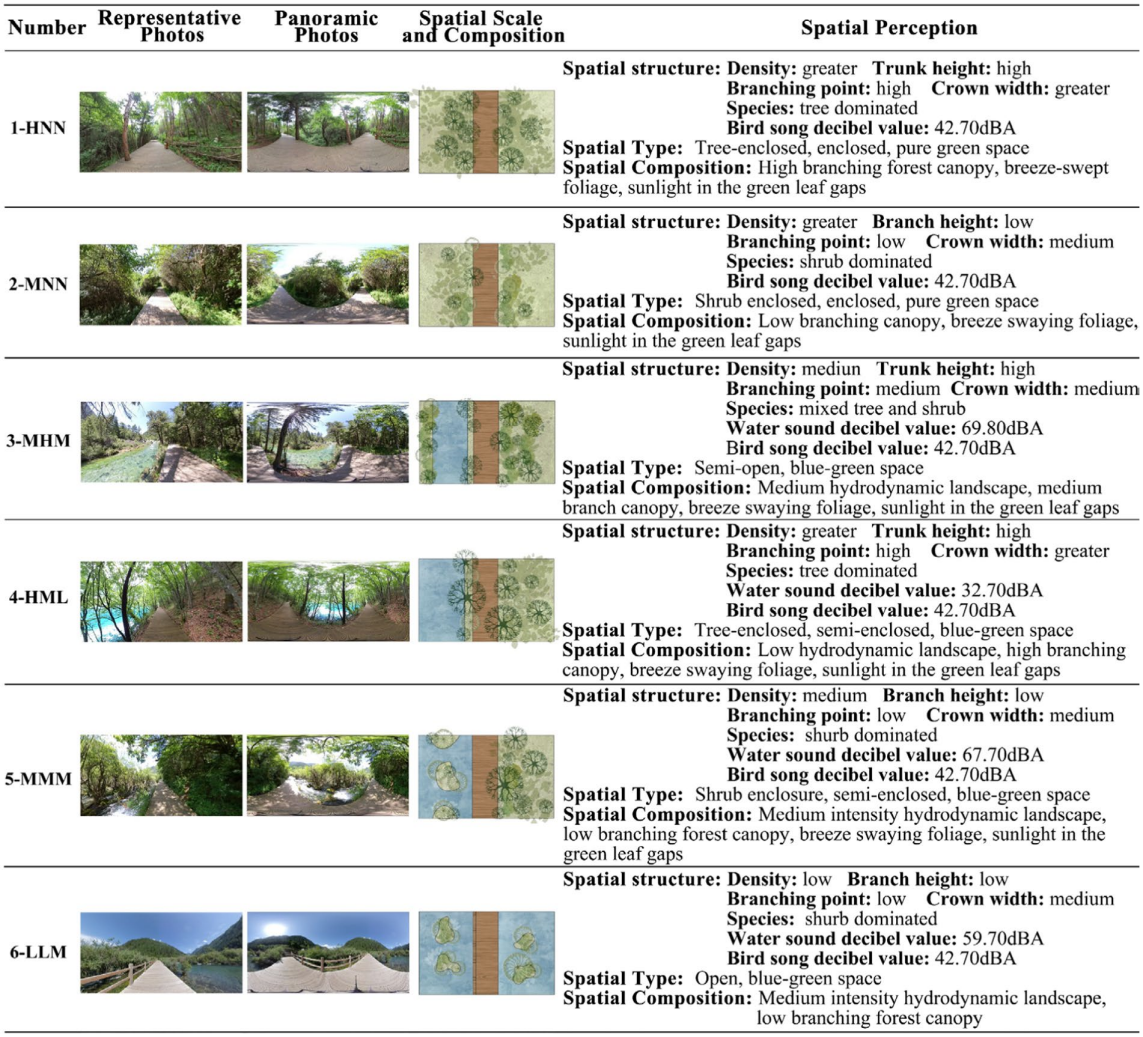
Description of the six categories of forest canopy and hydrodynamic landscapes.

**Figure 4:**
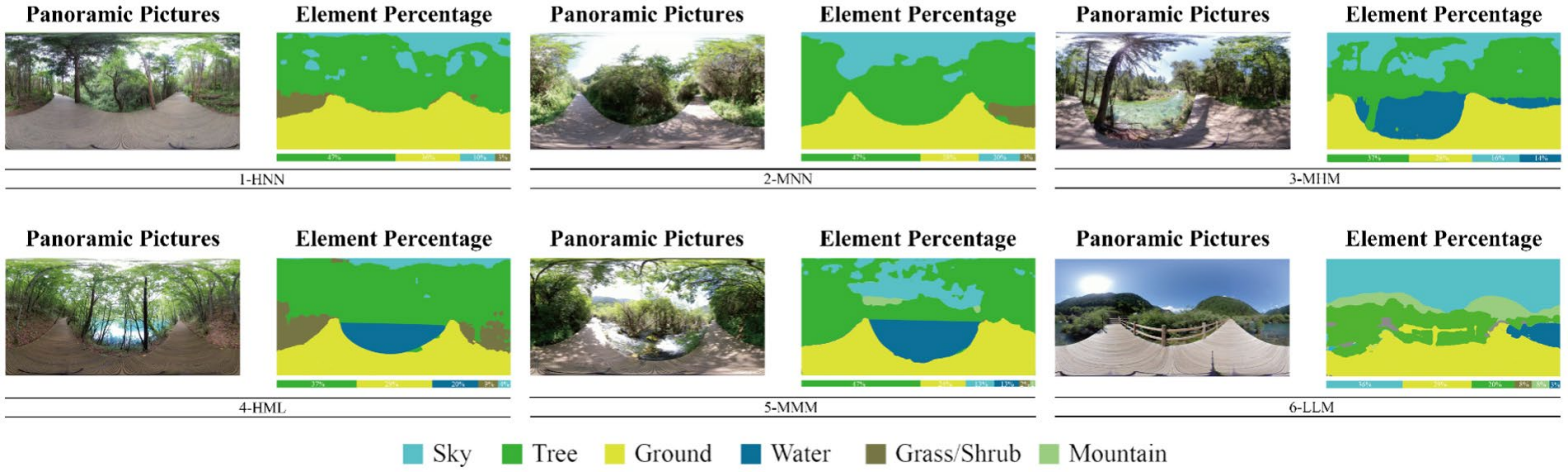
Panoramic images of the six scenarios and their semantic segmentation results.

The ADE20K model, developed by MIT, is a comprehensive collection used for semantic segmentation, and aids in understanding and identifying objects and structures within various scenes. Image segmentation was then conducted using deep learning techniques to determine the proportions of the six linear canopy landscapes, namely the sky, trees, sidewalks, water bodies, grass and bushes, and mountains. Based on the proportions of tree canopy landscapes (TREE/SKY) and water bodies (WATER/GROUND) and the sound pressure level or loudness of the six hydrodynamic landscape types, the landscapes were categorized into six classes: High-None-None (HNN; with ratio values of 4.53, 0.00, and 0.00), Medium-None-None (MNN; with ratio values of 2.60, 0.00, and 0.00), Medium-High-Medium (MHM; with ratio values of 2.27, 0.52, and 69.80), High-Medium-Low (HML; with ratio values of 5.96, 0.31, and 32.70), Medium-Medium-Medium (MMM; with ratio values of 3.48, 0.31, and 67.70), and Low-Low-Medium (LLM; with ratio values of 0.55, 0.11, and 59.70). In this study, we used Adobe Audition to reduce the noise of raw audio data collected prior to the experiment and introduced A-rate weighting (A-weighted sound level simulates the frequency characteristics of the human ear for noise with an intensity of 55 dB or less; Figures 3, 4; Tables 1, 2).

**Table 1:** Semantic segmentation percentage results of the panoramic images for the six scenarios.

	1	2	3	4	5	6
SKY	10.07%	18.13%	15.71%	8.03%	11.82%	35.31%
TREE	45.60%	47.14%	35.62%	47.89%	41.08%	19.55%
GROUND	31.60%	26.99%	27.00%	24.67%	27.54%	25.21%
WATER	/	/	13.97%	7.60%	10.61%	2.78%
GRASS/SHURB	3.08%	2.59%	/	3.66%	/	/
MOUNTAIN	/	/	/	/	/	6.85%

**Table 2:** Definition of the six types of forest canopy landscapes alongside hydrodynamic landscapes.

	1	2	3	4	5	6
TREE/Sky	4.53	2.60	2.27	5.96	3.48	0.55
WATER/GROUD	0.00	0.00	0.52	0.31	0.39	0.11
WATER loudness	0.00	0.00	69.80	32.70	67.70	59.70
TYPE	HNN	MNN	MHM	HML	MMM	LLM

The minimum threshold for dividing the TREE/SKY gradient in this study is 2, defining a 0–2 range for low canopy landscapes, a 2–4 range for medium canopy landscapes, and a 4+ range for high canopy landscapes. The minimum threshold for delineating the WATER/GROUND gradient is 0.2, defining no water as 0, low water as 0–0.2, medium water as 0.2–0.4, and high water as 0.4 or more.

### 2.4 Study site

**Figure 5:**
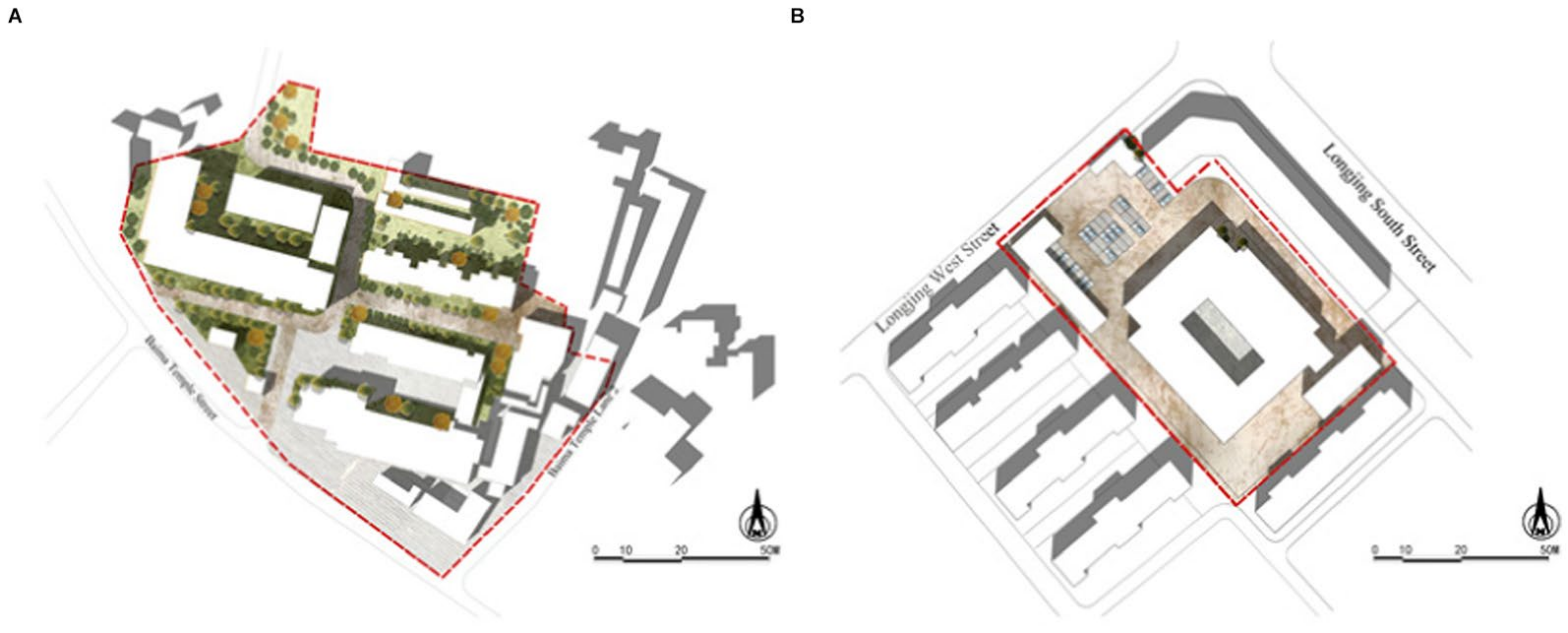
The study sites in this experiment. **(A)** Sichuan Province Forestry Central Hospital; **(B)** the 3rd People’s Hospital of Wuhou, Chengdu.

The experiment was conducted at Sichuan Province Forestry Center Hospital, the 3rd People’s Hospital in Wuhou, Chengdu, and the Wenchuan Traditional Chinese Medicine Hospital. Two hospitals are located in urban areas of Chengdu, with green coverage rates of 44.36 and 1.89%, respectively (Figure 5).

### 2.5 Measurements

**Figure 6:**
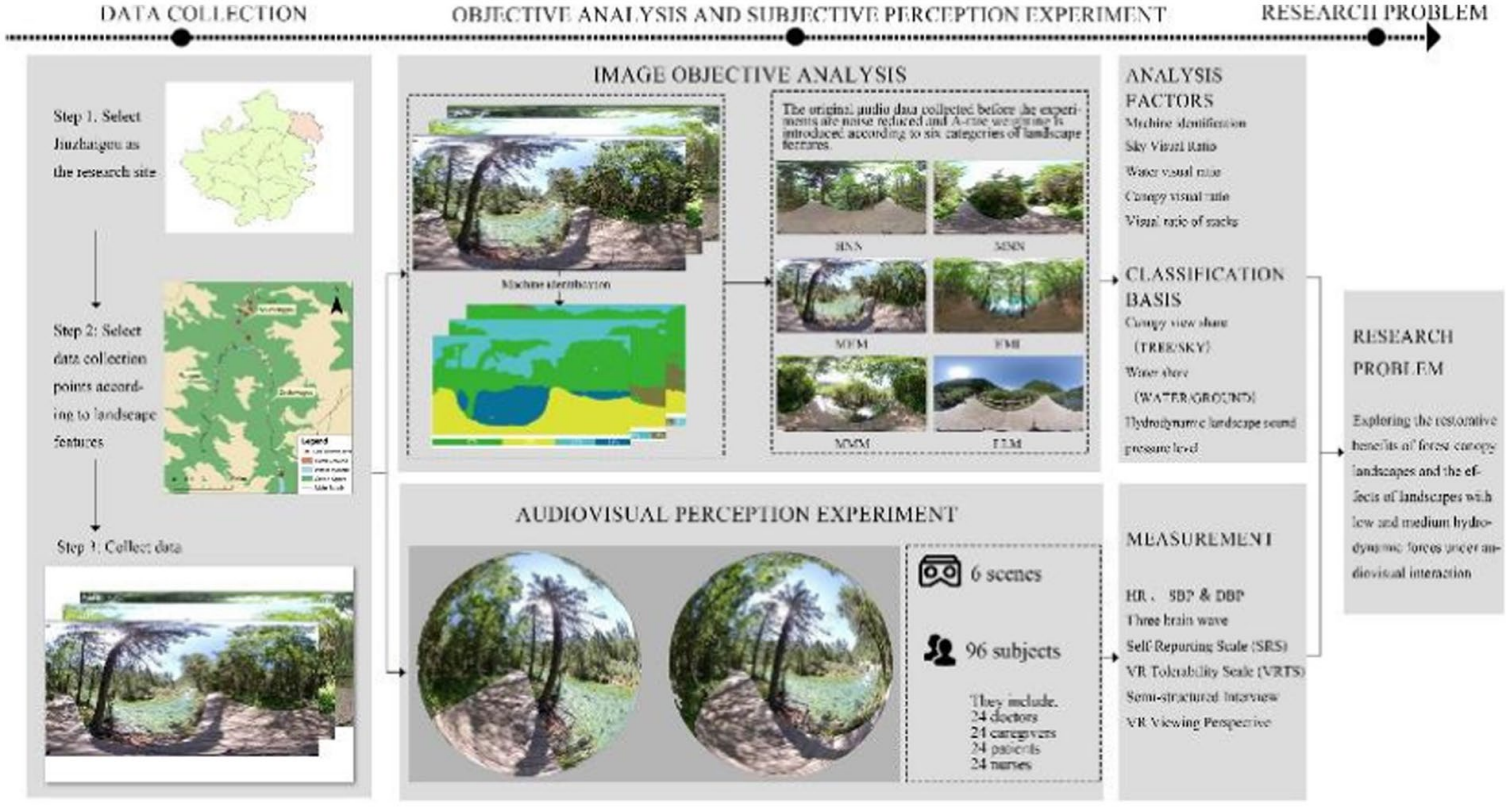
Workflow.

The physiological assessment data was collected by measuring the heart rate (HR), blood pressure (BP), and recording electroencephalogram (EEG). Psychological assessment data was collected by applying the Self-Report Scale (SRS), Anxiety Measurement Scale (AMS), VR Tolerability Scale (VRTS), and Semi-structured Interview. The observational method was used to record information on the landscapes that the subjects (Figure 6).

#### 2.5.1 Physiological measurement

Blood pressure, including Diastolic Blood Pressure (DBP) and Systolic Blood Pressure (SBP), is an indicator of the relaxation degree of the body (34). The heart rate is an indicator of physiological responses to physiological stimuli and psychological stress (35). Electroencephalography measures physiological responses in virtual reality (36, 37). Theta waves are a source of creativity and inspiration that influence a person’s attitudes, expectations, beliefs, and behaviors. Increased theta waves usually indicate a state of deep relaxation, in which inspiration emerges and creativity increases (38). Alpha waves are strongly related to stress relief. Increased alpha waves usually indicate a state of relaxation (39, 40). An increase in beta waves is generally associated with a state of alertness, while a decrease is associated with a state of sleepiness (41). This study applies the Wise Brain Instrument (Sichiray03, China) to measure changes in the participants’ brain waves. During measurements, a wireless device is connected to electrodes in the forehead, and the brain wave data is transmitted to a computer in real-time. The data is shown numerically, indicating the restoration degree of the participants (42).

#### 2.5.2 Psychological measurements

**Figure 7:**
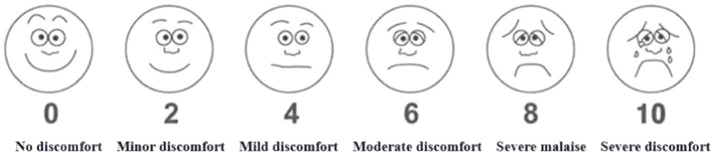
VR tolerability scale.

Previous research has shown that psychological recovery can be measured in five dimensions: restorative experiences, positive emotions, stress relief, cognitive physiological responses and landscape preferences (32, 43–45). All descriptions of the self-report scale in this study are adjudged to meet the claims of this study and the cognitive context of the subjects. The SRS consists of 14 items in five dimensions of restorative experiences, positive emotions, stress relief, cognitive physiological responses and landscape preferences and each item is rated from 1 to 7, where 7 indicate the highest level of psychologically restorative effects and 1 indicates the lowest level (Table 3). The total value of Cronbach’s a coefficient for the scale is 0.957, and each value of the five dimensions within the scale exceeds 0.7 (29). This indicates that the internal reliability of the evaluation items is quite remarkable. In addition, to control the effects of other variables due to population differences, we adopt the Anxiety Measurement Scale (AMS), a nonverbal image assessment scale that visually quantifies the subjects’ anxiety state (46). The scale is rated from 1 to 10, with 0 representing “no anxiety at all” and 10 representing “the most severe anxiety imaginable.” VR Tolerability Scale (VRTS), a non-verbal image assessment scale modified from the Face Pain Scale (FPS-R), is rating from 0 to10, with 0 representing “no discomfort” and 10 representing “the most severe discomfort imaginable” (Figure 7).

**Table 3:** Self-reporting scale.

Dimension	Item	Scale
Restorative experiences	Do you think your presence in these landscapes will help you to feel better/help you to feel restored?	1 2 3 4 5 6 7
	Do you think that if you had the opportunity to see the landscape every day during your stay in hospital it would help you to feel better/ help you to feel recovered?	1 2 3 4 5 6 7
	Do you think that being in these landscapes will help you to leave your worries behind for a while?	1 2 3 4 5 6 7
	Do you feel that these landscapes help you to take a break from your everyday life?	1 2 3 4 5 6 7
Positive emotions	Do you find that being in these landscapes gives you a little more energy?	1 2 3 4 5 6 7
	Do you find it more enjoyable to be in these landscapes?	1 2 3 4 5 6 7
Stress reduction	Do you feel that these landscape settings help you to gain a sense of inner peace?	1 2 3 4 5 6 7
	Do you find it relaxing to be in a landscape environment?	1 2 3 4 5 6 7
	Do you think that being in a landscape environment helps you to feel less anxious or stressed?	1 2 3 4 5 6 7
Physiological dimension	Do you think you become a little more focused when you are in these landscape environments?	1 2 3 4 5 6 7
	Do you think being in these landscapes will help you feel less mentally tired?	1 2 3 4 5 6 7
	Do you think you can breathe a little easier in these landscapes?	1 2 3 4 5 6 7
Landscape preferences	Do you find the landscape settings you have seen attractive to you?	1 2 3 4 5 6 7
	Do you think you would like to stay in these landscapes and continue to enjoy them?	1 2 3 4 5 6 7

#### 2.5.3 Semi-structured interview

The post-test semi-structured interview contains the following three open-ended questions:

1. Which elements of the landscapes do the participants prefer?
2. What elements can contribute to the psychologically restorative benefits of a landscape?
3. Would you like anything to be added to the landscapes?

#### 2.5.4 Observational method

We synchronously recorded the participants’ expressions, posture and speech, as well as the video frame the participants paid the most attention to and the duration of their attention through the connected video with SteamVR (HTC VIVE Focus3, Taiwan). The playback progress bar on the computer display screen during the experiment recorded the duration of attention.

### 2.6 Experimental procedure

The experiment was performed during September–October in 2022 (over 45 days) from 9:00 am to 11:00 am and from 2:30 to 4:30 pm. It was conducted in empty wards in the inpatient buildings of the two hospitals with the support from all the departments. Each test was conducted under no external interventions occurred by negotiating with the head nurses. Consistent physical conditions were maintained throughout the experiment with noise below 25 dB, suitable lighting, temperature from 20 to 23°C, and relative humidity from 45 to 55%. We employed the professional version of HTC-VIVE (Taiwan) as the VR device. The device contains two positioners and a headset with the following parameters: refresh rate of 90 Hz, the maximum field angle of 110°; and resolution ratio of 2,880*1,600.The video broadcasting was smooth and clear. Moreover, the built-in Hi-Res-certified headphones had a high sound quality.

**Figure 8:**
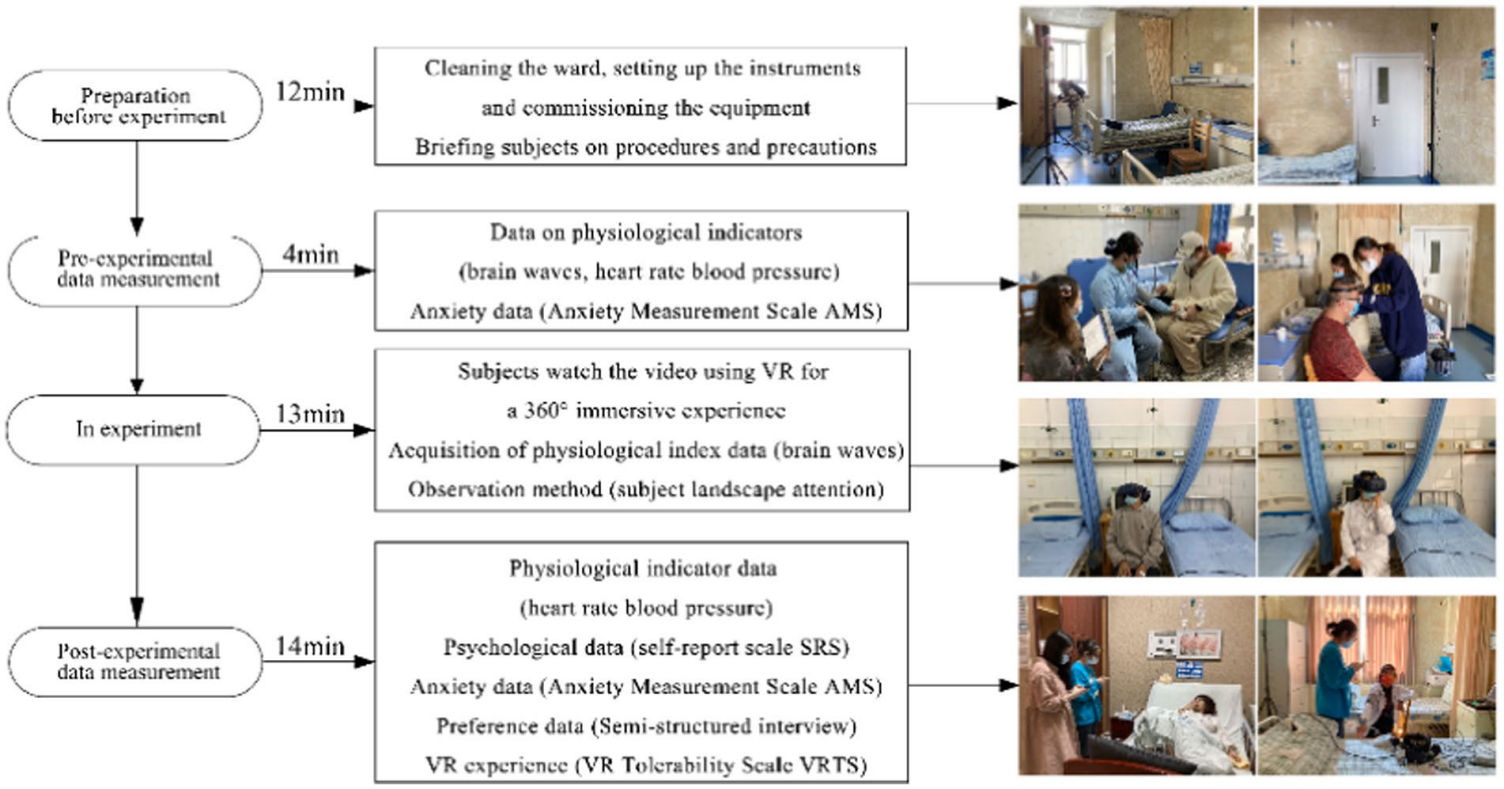
Experimental flowchart.

Before the experiment, the VR equipment was set up in an empty ward and debugged by the study Group. The subjects were individually guided in an orderly manner to the ward to conduct the test. The whole process lasted approximately 43 min (Figure 8).

First, the experiment and the apparatus were introduced to the subjects to relieve their stress. The subjects’ conditions of age, occupation, income, living environment, visit frequency, and green space preferences were recorded. The participants were then equipped with the brainwave scanner with the help of the members of the study group, who were also in charge of debugging the device to ensure proper signal transmission.

Second, participants’ blood pressure and heart rate were tested and the baseline measurements of the brainwave were collected for 80s. The participants were instructed to complete the pre-test AMS.

Third, the participants were seated facing the VR receiver, and the participants began to immersively experience six linear landscapes at 360°, including canopy landscapes and hydrodynamic landscapes. In consideration of the fact the experiment is conducted in the next day of one of the participants’ delivery, One participant gave birth the day before the test and was thus allowed to participate in the experiment lying against pillows in bed. Each landscape was experienced for 80 s (with bird songs added to the landscapes in the last 40 s). To avoid fatigue of attention, the participants were asked to close their eyes for 30 s at the end of each landscape experience. The participants could end the test quits at any time if they felt uncomfortable. When the participants were observing the landscapes through VR, the members of the study group recorded the subjects’ expressions, posture and speech, as well as the video frame that the participants paid the most attention to and duration of their attention.

After the VR experiences, the participants were assisted in removing the VR device and asked to rest for 1 min to measure heart rate and blood pressure, They were instructed to fill out the SRS, AMS and VRTS. Based on the age, health, and physical load of the participants, as well as the possible discomfort and negative emotions resulting from repeatedly taking the brainwave scanner on and off (even if the scales could be filled by the study group on behalf of the participants), the participants were asked to complete the SRS only once after the EEG instrument was removed at the end of all the tests, rather than at the end of each test. A semi-structured interview was then used to conduct to ask subjects about their favorite spaces, elements of the landscape and expectations.

### 2.7 Statistical analysis

The sample size for the experiment was 96. To verify whether the data conformed to a normal distribution, we conducted the Shapiro–Wilk test on all sample data. The results showed that the *p*-values were all greater than 0.05. Therefore, we accepted the null hypothesis and concluded that the data from the experimental conformed to a normal distribution. Based on this, we used parametric statistical methods for subsequent analysis. SPSS 26.0 was used for the data processing and paired *t*-test to analyze and compare the differences of the mean physiological and psychological indicators values of each audiovisual test in the six landscapes with those of the baseline. The differences in the mean values were used for the range ratings, Analysis of variance (ANOVA) was employed to analyze and compare the between-group differences in the physiologically and psychologically restorative effects of the four groups before and after the audiovisual interaction tests in the six landscape types.

## 3 Results

### 3.1 Physiological results

#### 3.1.1 Heart rate and blood pressure

**Figure 9:**
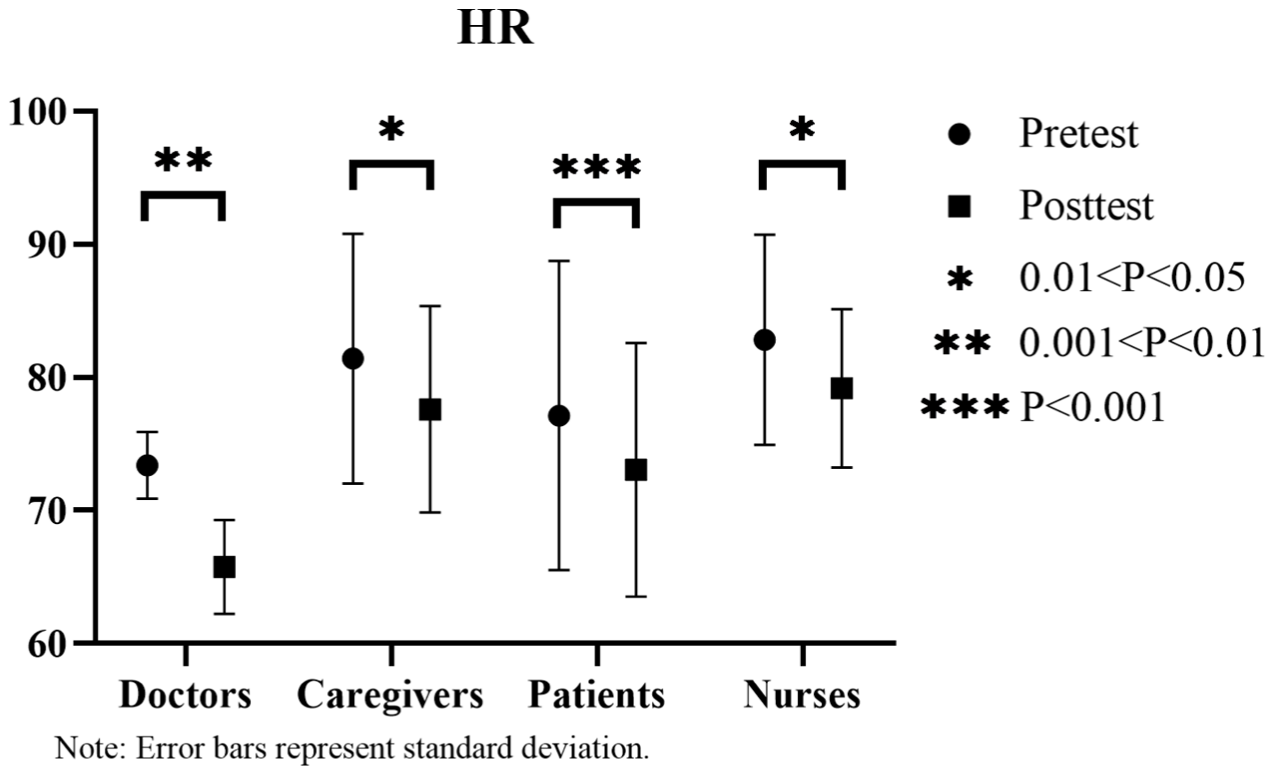
Changes in heart rate before and after the experiment.

From the pre- and post-experimental heart rate data, significant decreases in heart rates are observed among doctors ([73.375 ± 2.50] to [65.75 ± 3.53]; *p* < 0.05), caregivers ([81.4 ± 9.37] to [77.6 ± 7.74]; *p* < 0.05), patients ([77.13 ± 11.62] to [73.06 ± 9.54]; *p* < 0.05), and nurses ([82.83 ± 7.90] to [79.17 ± 5.96], *p* < 0.05). Among them, doctors exhibit the most significant decrease in heart rate values (7.625; Figure 9).

**Figure 10:**
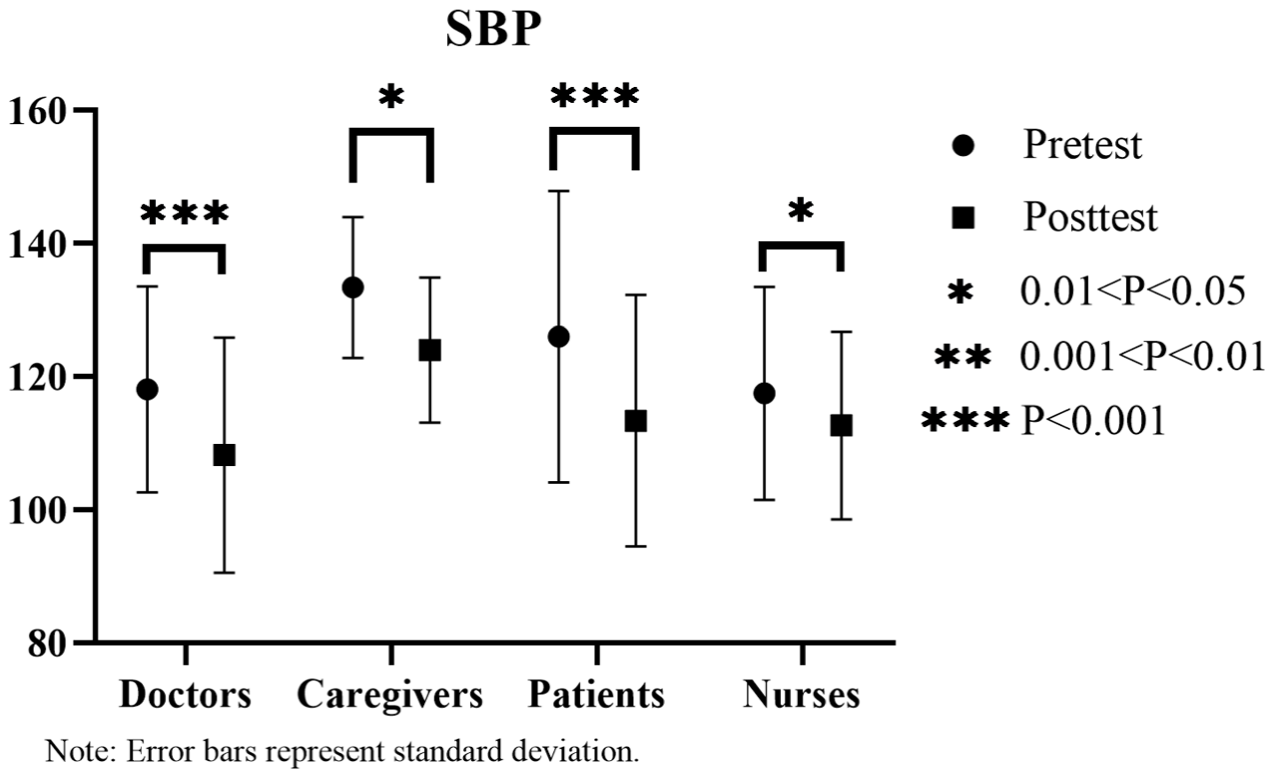
Changes in systolic blood pressure (SBP) before and after the experiment.

**Figure 11:**
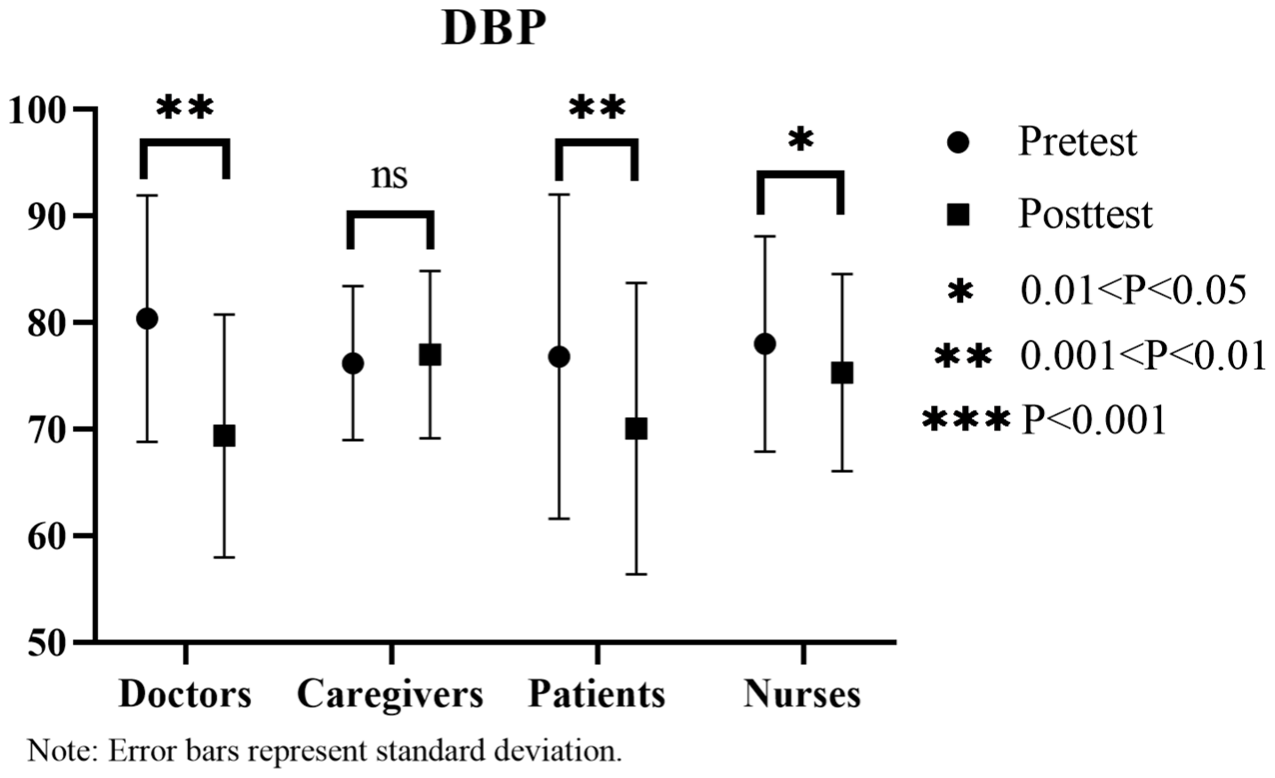
Changes in Diastolic Blood Pressure (DBP) Before and After the Experiment.

From the pre- and post-experimental blood pressure data, significant decreases are observed among doctors, patients, and nurses. In particular, diastolic blood pressure decreases from [80.86 ± 11.48] to [70.86 ± 10.59], and systolic blood pressure decreases from [80.375 ± 10.82] to [69.375 ± 10.65] (*p* < 0.05) among doctors. For patients, diastolic blood pressure decreases from [76.81 ± 15.22] to [70.06 ± 13.68], and systolic blood pressure decreases from [126 ± 21.90] to [113.38 ± 18.89] (*p* < 0.05). For nurses, diastolic blood pressure decreases from [78 ± 10.12] to [75.33 ± 9.25], and systolic blood pressure decreases from [117.5 ± 15.99] to [112.67 ± 14.07] (*p* < 0.05). Caregivers’ diastolic blood pressure does not exhibit any significant differences, while systolic blood pressure decreases significantly (diastolic blood pressure [76.2 ± 7.22] to [77 ± 7.85], *p* > 0.05; systolic blood pressure [133.4 ± 10.57] to [124 ± 10.89], *p* < 0.05). Notably, patients exhibit the most significant decrease in diastolic blood pressure (12.62; Figures 10, 11).

#### 3.1.2 Results of within-group brain wave control tests for audiovisual perception in the six landscapes with varying bird songs as a variable

**Figure 12:**
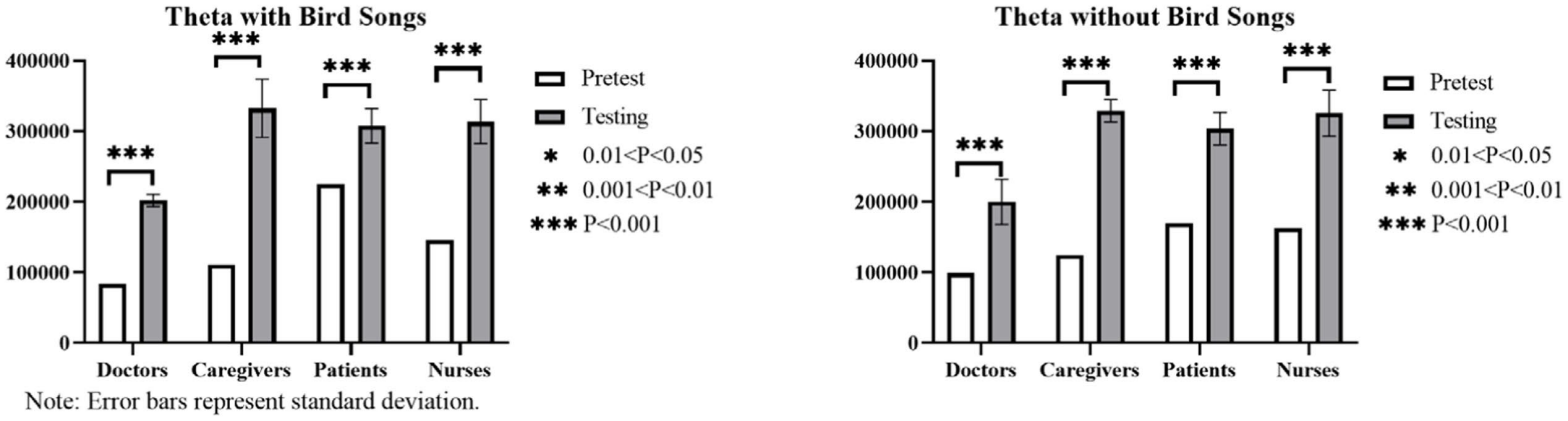
Histogram of the within-group brain wave control test results (theta waves) for audiovisual perception in the six linear canopy landscapes with varying bird songs as a variable.

**Figure 13:**
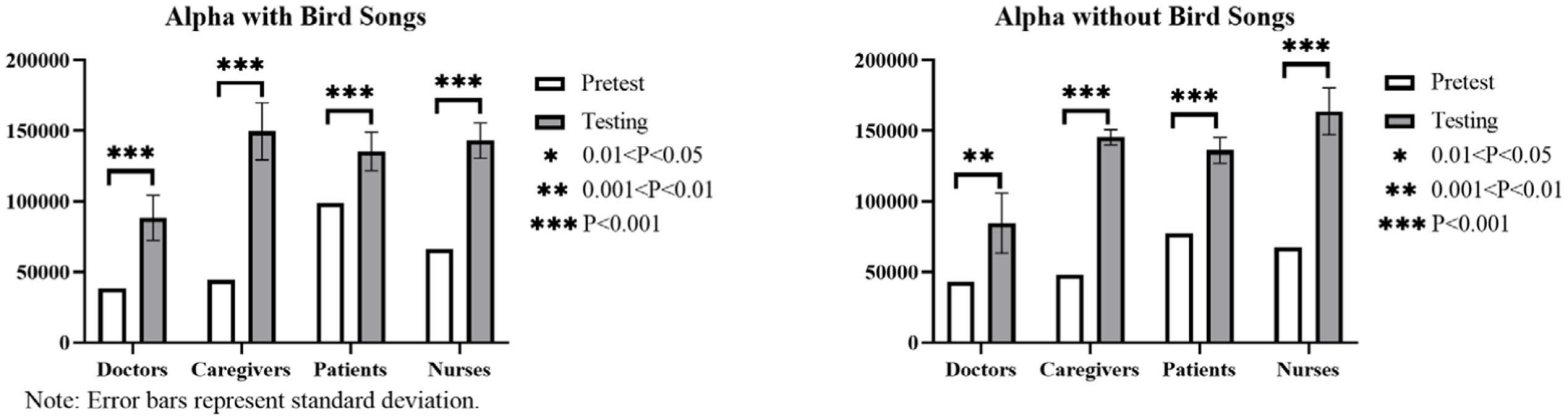
Histogram of the alpha wave control test results for audiovisual perception in the six linear canopy landscapes with bird songs as a variable.

**Figure 14:**
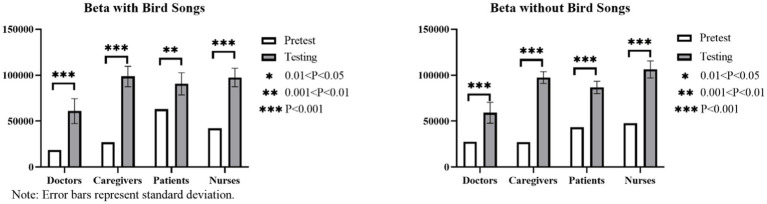
Histogram of the beta wave control test results for audiovisual perception in the six linear canopy landscapes with bird songs as a variable.

Based on the results of the paired *t*-test and the mean difference results described in Figures 12–14, a notable increase in three brain waves is observed across all four groups in the six linear canopy landscapes without bird songs (*p* < 0.05). These results can be ranked in descending order of the range of for the three waves as follows: caregivers (θ increases by 204, 180, α increases by 97,482, β increases by 70,191); nurses (θ increases by 163,003, α increases by 96,686, β increases by 58,607); patients (θ increases by 134,583, α increases by 58,709, β increases by 43,332); and doctors (θ increases by 100,856, α increases by 41,441, β increases by 31,519). When bird songs are introduced into the landscapes, the results indicate the following rank order of brain wave increases: caregivers (θ increases by 221,463, α increases by 105,066, β increases by 71,628); nurses (θ increases by 167,814, α increases by 76,907, β increases by 54,969); doctors (θ increases by 118,029, α increases by 49,994, β increases by 42,287); and patients (θ increases by 81,941, α increases by 36,374, β increases by 27,608). Thus, the addition of bird songs has the most pronounced influence on the brain waves of caregivers, followed by nurses.

The one-way ANOVA results reveal a low range of the three brain waves among patients, doctors, and caregivers (*p* > 0.05), indicating no significant difference in EEG data across these groups when bird songs are introduced. For nurses, the addition of bird songs does not significantly affect θ and β (p > 0.05), but has a notable impact on α (*p* < 0.05).

#### 3.1.3 Results of between-group texts on brain waves of audiovisual interactive perception in the six linear canopy landscape types with bird songs as a variable

**Figure 15:**
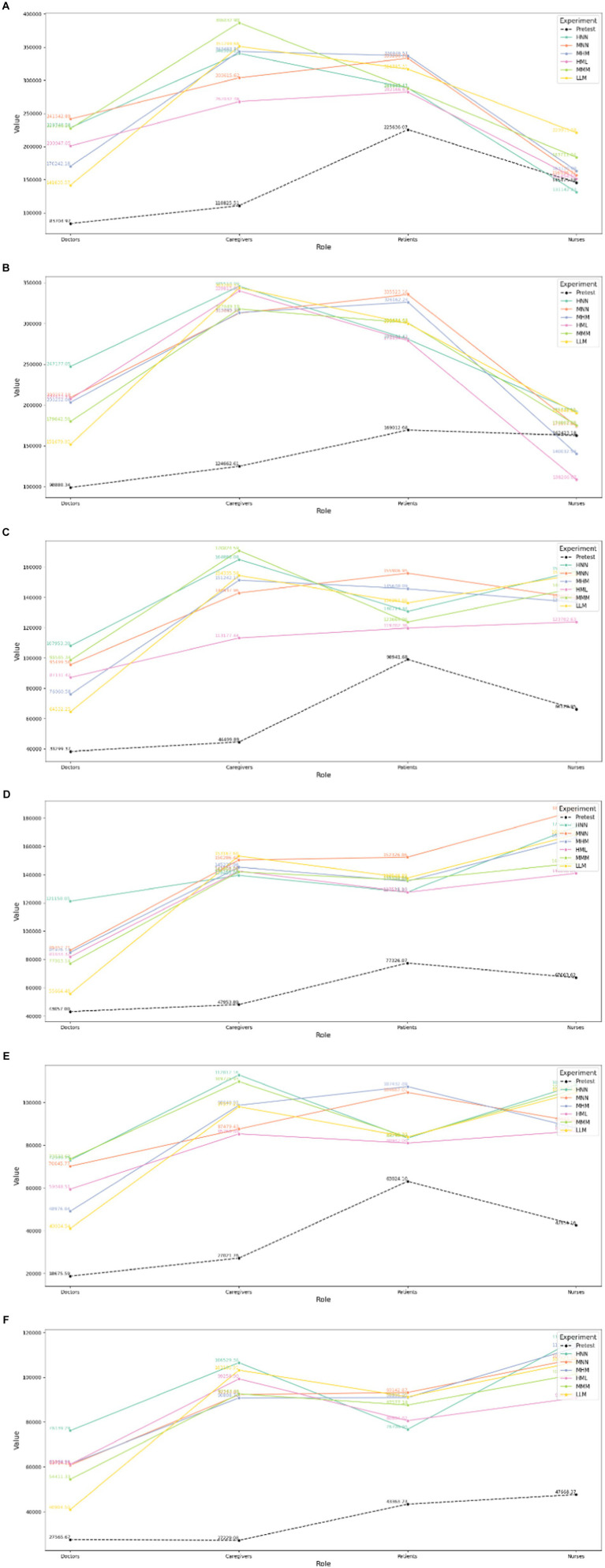
Results of between-group control texts on brain waves of audiovisual interactive perception in the six linear canopy landscapes with bird songs as a variable. Comparison of θ values for different subjects **(A)** with and **(B)** without bird songs. Comparison of α values for different subjects **(C)** with and **(D)** without bird songs. Comparison of β values for different subjects **(E)** with and **(F)** without bird songs.

The maximum mean values of theta, alpha and beta waves for the four groups when bird songs are not added are observed in the following landscapes, respectively: HNN, HNN, and HNN for doctors; HNN, LLM, and LLM for caregivers; MNN, MNN, and MNN for patients; and HNN, MNN, and HNN for nurses (Figure 15). When bird songs are added, the corresponding results are as follows: MNN, HNN, and MMM for doctors; MMM, MMM, and HNN for caregivers; MHM, MNN, and MHM for patients; and LLM, HNN, and HNN for nurses.

### 3.2 Results of psychologically restorative effects

#### 3.2.1 SRS

The SRS values for the four groups range between 5.39 and 6.30 (Table 4). The highest values for each item are observed in the caregivers group, with the value ranges for restorative experiences, positive moods, stress relief, physically cognitive response, and land-scape preferences determined as6.25 ± 0.75, 6.30 ± 0.27, 6.20 ± 0.84, 6.30 ± 0.57, and 6.20 ± 0.57. The results of the one-way ANOVA show that there are no significant between-group differences in the values of each item (*p* > 0.05) for the four groups.

**Table 4:** SRS results for the four groups.

Subjects	Doctors	Caregivers	Patients	Nurses
Average	5.75	6.25	5.79	5.50
Standard deviation	0.866	0.750	1.315	1.095
Ratings	3	1	2	4
Positive emotion				
Average	5.56	6.30	5.32	5.42
Standard deviation	0.821	0.274	1.636	1.242
Ratings	2	1	4	3
Stress relief				
Average	5.81	6.20	5.71	5.39
Standard deviation	0.753	0.837	1.437	1.182
Ratings	2	1	3	4
Physiological cognitive response				
Average	5.71	6.30	5.43	5.61
Standard deviation	0.950	0.570	1.207	0.976
Ratings	2	1	4	3
Landscape preference				
Average	5.66	6.20	6.04	5.92
Standard deviation	0.694	0.758	1.407	1.320
Ratings	4	1	2	3
Total score rating				
Average	28.49	31.25	28.29	27.83
Standard deviation	0.095	0.050	0.286	0.214
Ratings	2	1	3	4

#### 3.2.2 Anxiety measurement results

**Figure 16:**
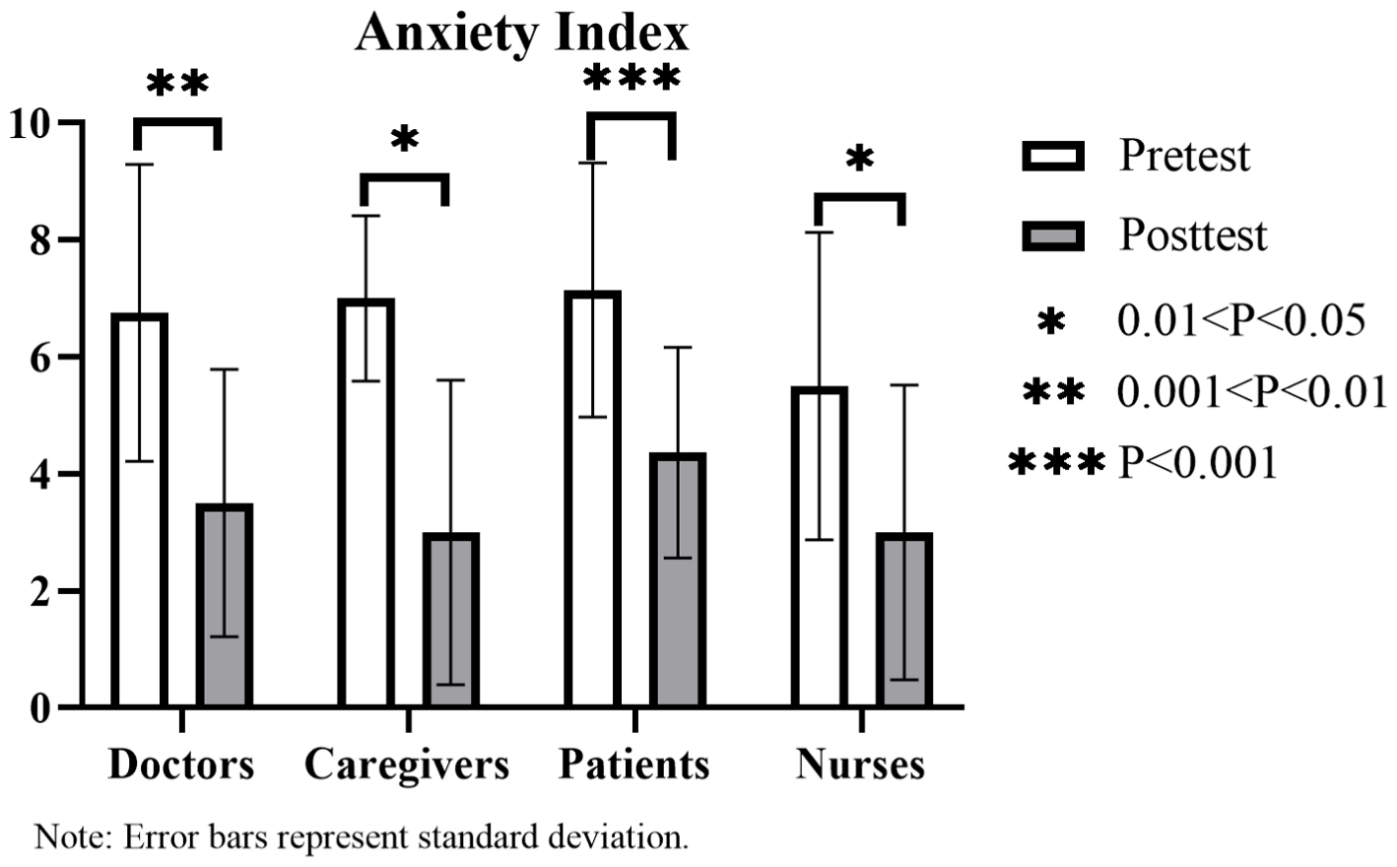
Changes in the anxiety level of the four groups.

According to the one-way ANOVA results, there are no significant differences in pre-test anxiety level values among the four groups (*p* > 0.05). The arithmetic mean values exceed 5.50, with the values ranges for patients, caregivers, doctors, and nurses determined as 7.14 ± 2.17, 7.00 ± 1.41, 6.75 ± 2.5, and 5.50 ± 2.63. Figure 16 depicts the paired *t*-test results, indicating a significant reduction in anxiety after the experiment in the four groups. The four groups can be rated from the greatest to the least decline in anxiety degree as follows: caregivers, doctors, patients, and nurses.

## 4 Discussion

### 4.1 Psychologically restorative effects of canopy landscapes and those with varying hydrodynamic forces for hospital populations

The SRS results indicate that linear canopy landscapes and landscapes with low and medium hydrodynamic forces exert significant psychologically restorative effects on hospital populations, with the greatest effects observed in caregivers who also have the highest preferences for such landscapes. It is argued that the natural green space providing audiovisual interactive perception can serve as a therapeutic space, especially for caregivers who need to care for and worry about patients.

According to the AMS results, all four groups exhibit a high baseline level of anxiety before the experiment. This is consistent with the testing performed prior to the experiment and interviews with a wide range of hospital populations. Each of the four groups was experiencing anxiety due to particular issues. After the experiment, the level of anxiety was significantly reduced for all groups, with the greatest reduction observed for care-givers, followed by doctors. Patients, on the other hand, experienced the minimum decline in anxiety. This may be because patients, the core population in hospitals, are influenced by factors such as disease perception, physical conditions, treatment, and the hospital environment (47). Thus, patients have the highest baseline level of anxiety and the lowest reduction of anxiety level. In contrast, the anxiety of the caregivers mainly comes from their mental state, related to caring and worrying about the patients and feelings of depression. According to Winnie’s study, trivial affairs in daily life have the greatest impact on mental state, rather than significant life events (48). When accompanying and attending to patients, caregivers are faced with two key anxiety sources, namely, an increase in daily trivial affairs and the major event of relatives being hospitalized. This consequently brings caregivers a high level of psychological stress. Stress recovery theory proposes that the natural environment has remarkable restorative benefits for relieving stress (31). The restorative benefits of the natural environment for relieving psychological stress contribute to a significant decrease in the anxiety levels of the caregiver groups.

### 4.2 Physiologically restorative effects of canopy landscapes and those with varying hydrodynamic forces for hospital populations

According to the paired *t*-tests for blood pressure and heart rate, both the linear forest canopy landscapes and the landscapes with low and medium hydrodynamic forces possess great physiological recovery benefits for different populations in hospitals, among which the greatest benefits observed for doctors and patients. As for patients in particular, the physiologically restorative benefits may also be greater than the psychologically restorative benefits. According to brain wave data, landscapes with low hydrodynamic forces are less restorative in hospital settings. This is related to the fact that hospital populations are faced with fear and stresses brought by diseases and death, and have desire for vitality (47, 49–51). Waterscapes with medium hydrodynamic forces are more likely to exert psychological effects on hospital populations with more vibrant and lively features, especially in the group of patients.

Swaying and light-transmitting verdant foliage and landscapes with medium hydrodynamic forces under a green background can help doctors relax temporarily. This may be related to the occupational load and risks of doctors (52). Open water has relatively strong restorative benefits for caregivers and nurses. This may be because the stress of caregivers originates from mental problems (e.g., worry, anxiety, and fear) due to sick family members, physical fatigue owing to attending patients (also seen in the nurses group), and the self-repression of needs and desires. Thus, open water even with less green space can help to relieve their emotional burden and get a sense of inner peace, relaxation, and power. Large waterscapes, such as lakes and rivers, have significant restorative benefits, creating a wide view and sense of awe for visitors (53).

Previous research reports that high branching points or increasing canopy densities can add to the aesthetic features (54). In contrast, this study finds that canopy landscapes with branches of low and medium canopy width are relatively more restorative for the group of patients. This may be because tall trees and water bodies with wide views may bring patients a vague sense of past one’s prime in life, oppression and insignificance rather than a sense of power and majesty. On the contrary, the landscapes with low branch canopy width can trigger a sense of prosperity and intimacy. In addition to the increasing lively and vital atmosphere, the landscapes with medium hydrodynamic forces can also bring visitors a bright, lively, positive and pleasant feeling (55). Thus the waterscapes with medium hydrodynamic forces in green space can also provide a sense of vitality.

### 4.3 Comparison of physiological benefits for hospital populations in linear canopy landscapes and varying hydrodynamic landscapes with and without bird songs

The brain wave test results indicate that there are no significant differences in physiological restorative benefits among the groups of patients, doctors and caregivers in the presence or absence of bird songs. Only the alpha wave values of nurses show significant fluctuations. This may be because the perception of bird sounds as a symbol of vitality and well-being may depend on participants’ existing connections to nature (56). According to the results of the semi-structured interviews, the nurse’s group is the most exposed to nature among the four groups. Under the cocktail party effect (57) on auditory attention, greater benefits of bird songs are observed for nurses. Moreover, due to their relatively lower level of anxiety, nurses are relatively more sensitive to low and medium-level auditory stimuli (sound level from 30 to 50 dB) compared to the other three groups, who are in relatively higher states of anxiety and stress over long periods of time. However, this difference in sensitivities is small. In fact, the nurses group also exhibits high anxiety. Thus, when people are under high stress for a long period, their sensitivity to the outside world can be reduced.

### 4.4 Scene preferences based on steam VR screens and interviews

**Figure 17:**
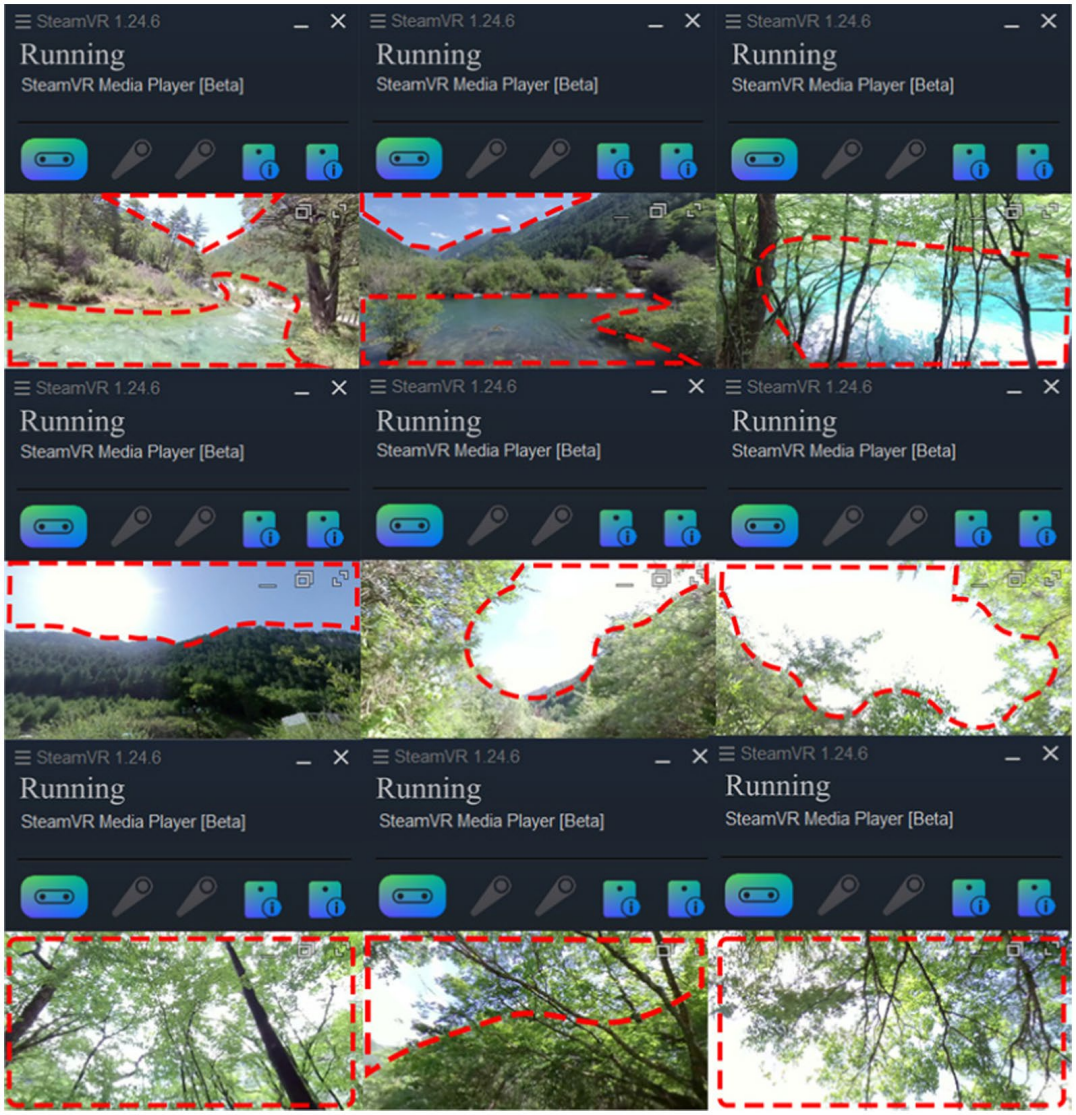
Hydrodynamic landscape Images, Sky Images and Forest Canopy Landscape Images.

Scholars have reported that people focus more frequently on their preferred landscape element compared to other landscape elements. The more frequently these elements occur, the more charming (58–60) and restorative (61, 62) the landscapes can become. Through the connected screen of Steam VR, it can be observed that the subjects paid more attention to the scenes of hydrodynamic landscapes, the scenes of the sky and ones of light-transmitting green foliage (Figure 17). In addition, post-test interviews revealed that due to their inner anxiety, fear of illness and death, and a desire for life, hospital populations, patients (particularly those suffering from serious diseases such as cancer) show a preference for landscapes with small animals or even young children, as well as the resistance to tall and thick tree trunks. Swaying and light-transmitting verdant foliage and landscapes with medium hydrodynamic forces in a green background can help doctors relax and gain a sense of hope. This demonstrates once again the significance of applying canopy landscapes and landscapes with low and medium hydrodynamic forces as therapeutic landscapes, as well as the applicability of audiovisual experience as an adjunct therapy for certain diseases.

## 5 Summary and perspective

This study investigates the restorative benefits of different linear canopy landscapes and the landscapes with low and medium hydrodynamic forces for hospital subgroups through audiovisual perception. It is confirmed that both the linear canopy landscapes and landscapes with low and medium hydrodynamic forces have positive effects on adjunct therapy. Under a green space background with light-transmitting swaying foliage, landscapes with medium hydrodynamic forces have the greatest restorative benefits for the group of doctors. Canopy landscapes with low and medium branch canopy width are more likely to convey psychologically restorative effects on the group of patients with more vibrant and lively features. Open water even in the background of green space can help the caregivers and nurses get a sense of inner peace. For the hospital population as a whole, there is no significant difference in physiologically restorative benefits in the presence or absence of bird songs.

The selection of participants in this study considered the participants’ ages and the experiment duration to ensure the representativeness of the sample. However, this work still has some limitations. For example, patients who are almost completely deprived of the opportunity to have a long-distance travel are not included in the study. Moreover, the videos collected in the study mainly feature natural landscapes and exclude artificial landscape elements. Future research can further compare the restorative benefits between the natural landscapes and artificial ones by taking the linear forest canopy landscapes and the landscapes with low and medium hydrodynamic forces as research objects. Patients suffering from cancer and even terminal ones may have a great desire for green spaces with artificial elements and features in a way beyond our imagination. Thus, it is of significance to pay more attention to the construction of therapeutic landscapes based on the benefits for the green equity, hygiene, health and well-being of patients.

## Data Availability

The original contributions presented in the study are included in the article/supplementary material, further inquiries can be directed to the corresponding authors.
